# Insights into the Uses of Traditional Plants for Diabetes Nephropathy: A Review

**DOI:** 10.3390/cimb44070199

**Published:** 2022-06-29

**Authors:** Haleema Shahin D. H., Rokeya Sultana, Juveriya Farooq, Tahreen Taj, Umaima Farheen Khaiser, Nader Sulaiman Ayyt Alanazi, Mohammed Kanan Alshammari, Mohammad Nazal Alshammari, Firas Hamdan Alsubaie, Syed Mohammed Basheeruddin Asdaq, Abdulmueen A. Alotaibi, Abdulrhman ahmed Alamir, Mohd. Imran, Shahamah Jomah

**Affiliations:** 1 Department of Pharmacology, Yenepoya Pharmacy College & Research Centre, Yenepoya (Deemed to be University) Mangaluru, Karnataka 575018, India; faryasmin79@gmail.com (H.S.D.H.); mhospital@gmail.com (J.F.); qualityasdaq@gmail.com (T.T.); basheer_1@rediffmailc.om (U.F.K.); 2 Department of Pharmacognosy, Yenepoya Pharmacy College & Research Centre, Yenepoya (Deemed to be University) Mangaluru, Karnataka 575018, India; 3 Department of Pharmacy, King Salman Specialist Hospital, Hail 55436, Saudi Arabia; ghar-11@hotmail.com; 4 Department of Pharmaceutical Care, Rafha Central Hospital, North Zone, Rafha 76312, Saudi Arabia; ii_kanan101@outlook.com; 5 Faculty of Pharmacy, Northern Border University, Rafha 91911, Saudi Arabia; mhamd.n130@gmail.com (M.N.A.); aafros19@gmail.com (F.H.A.); 6 Department of Pharmacy Practice, College of Pharmacy, AlMaarefa University, Dariyah, Riyadh 13713, Saudi Arabia; 7 Department of Anaesthesia Technology, College of Applied Sciences, AlMaarefa University, Dariyah, Riyadh 13713, Saudi Arabia; aotaibi@mcst.edu.sa; 8 Department of Emergency Medicine, Prince Sultan Military Medical City, Riyadh 12233, Saudi Arabia; a.almear@gmail.com; 9 Department of Pharmaceutical Chemistry, Faculty of Pharmacy, Northern Border University, Rafha 91911, Saudi Arabia; imran.pchem@gmail.com; 10 Pharmacy Department, Dr.Sulaiman Al-Habib Medical Group, Riyadh 11372, Saudi Arabia; shahama.joma@gmail.com

**Keywords:** diabetic nephropathy, streptozotocin, antioxidant, medicinal plants

## Abstract

Diabetic nephropathy (DN) is a serious kidney illness characterized by proteinuria, glomerular enlargement, reduced glomerular filtration, and renal fibrosis. DN is the most common cause of end-stage kidney disease, accounting for nearly one-third of all cases of diabetes worldwide. Hyperglycemia is a major factor in the onset and progression of diabetic nephropathy. Many contemporary medicines are derived from plants since they have therapeutic properties and are relatively free of adverse effects. Glycosides, alkaloids, terpenoids, and flavonoids are among the few chemical compounds found in plants that are utilized to treat diabetic nephropathy. The purpose of this review was to consolidate information on the clinical and pharmacological evidence supporting the use of a variety of medicinal plants to treat diabetic nephropathy.

## 1. Introduction

Diabetes mellitus is a set of metabolic disorders marked by hyperglycemia due toa defect in insulin secretion or insulin action or both. In 2021, as per the International Diabetic Federation, approximately 10.5% (537 million people) of adults aged between 20 to 79 years were affected with diabetes [1,2]. By 2030, it is expected that this number will rise to over 643 million people. The incidence rate of diabetic nephropathy (DN) is high in the first 10 to 20 years after the onset of diabetes (three% per year) [3].

Recent efforts to analyze the usage of a variety of medicinal plants as a cure for various ailments, including diabetes, have relied mostly on field surveys. The aim of the paper is to provide a review of the literature on the application of medicinal herbs to treat DN.

## 2. Pathogenesis of Diabetic Nephropathy

Hyperglycemia is a major risk factor for DN [4]. The pathophysiology of DN also involves the increased production of lipid peroxides, active carbonyl compounds and free radicals leading to oxidative stress and no enzymatic protein glycosylation. Antioxidant enzymes play a role in protecting cells and tissues from causing damage. The variation in the generation of free radicals and antioxidants is found to be the main reason for the development of disease.

**Figure 1:**
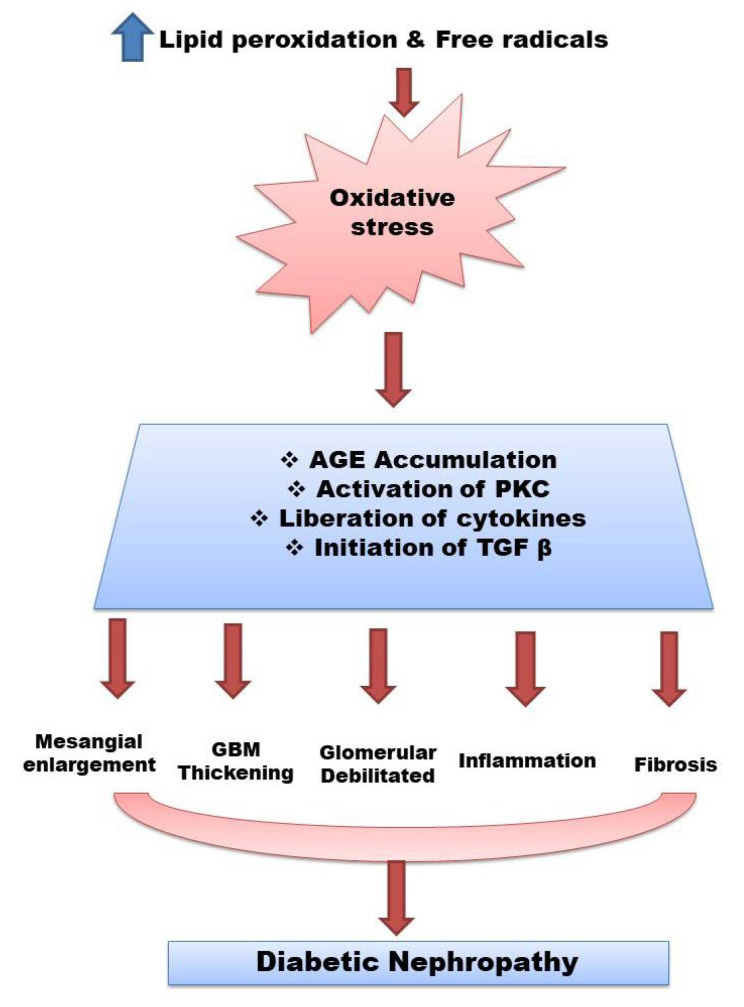
Pathophysiology of diabetes nephropathy. Abbreviations: Advanced glycosylated end products (AGEs), glomerular basement membrane (GBM), protein kinase C (PKC), transforming growth factor beta(TGF-*β*).

Nephropathy is caused primarily by the interaction of hemodynamic and metabolic systems. Metabolic pathways are also activated within the diabetic kidney, resulting in advanced glycosylated end products (AGEs) deposition, protein kinase C (PKC) activation, renal polyol synthesis, and enhanced oxidative stress. As a result, several cytokines and growth factors are activated. These processes eventually lead to changes in the renal histology of glomeruli, that is, mesangial expansion, glomerular basement membrane (GBM) thickening and glomerular sclerosis [5,6,7] (Figure 1).

## 3. Medicinal Plants and Diabetic Nephropathy

Natural remedies have received much interest in terms of medical research in recent years due to their capacity to heal a variety of ailments. Despite huge improvements in the pharmaceutical drug industry over the last two decades, the number of medications that can slow the progression of diabetes nephropathy is still restricted. Herbal treatments are popular because of their accessibility, cost effectiveness, lesser side effects, and high tolerance. Table 1 represents the list of a few medicinal plants and their action on DN.

### 3.1. Ginger

Often known as *Zingiber officinale*, Zingiberacea is the most commonly used herbal supplement in the world. Patients seek therapy from it for a variety of ailments, though it is commonly utilized for culinary purposes. Many analytical methods identify approximately 115 components in fresh and dried ginger. Fresh ginger contains a large amount of gingerol. Some of the terpene components present in ginger includes *β*-bisabolene, zingiberene, *α*-farnesene, *β*-sesquiphellandrene and *α*-curcumene, which are also identified to be the main active components of ginger essential oil [7]. Plants possess many medicinal uses including anti-diabetic, nephroprotective, hepato-protective, anti-cancer, antioxidant, analgesic, immunomodulatory, and anti-inflammatory properties [21,22,23].

In ayurveda, ginger is recommended for its traditional properties such as cardio protection, appetite stimulant, anti-asthmatic, for constipation, pain reduction, for normalization of blood circulation etc. [24].

A study was performed by Al Hroob et al. [8] on an experimental animal where a rat was given an intraperitoneal injection of streptozotocin to induce diabetes. Treatment was given orally with 400 or 800 mg/kg/day with *Z. officinale* extract for 45 days. A biomarker was analyzed after the treatment, and it was found that there was an elevated level of blood urea, nitrogen urea, urine albumin, and serum creatinine in the diabetic animal and it was found to be reversed in the animals treated with *Z. officinale.* There was a significantly lower lipid level and blood glucose level in the extract treated animals, but rats induced with diabetes showed an alleviation of blood glucose and lipid level. This result is supported by findings from histopathology where treatment with *Z. officinale* reduces histological alteration in the kidneys of diabetes induced rats. There was a remarkable elevation in the level of pro-inflammatory cytokines, Cytochrome c, malondialdehyde, and protein carbonyl in chronic hyperglycemia induced diabetic rats which were reversed by Z. *officinale* treatment. According to the findings of this study [8], *Z.officinale* rhizome extract protects against diabetes-related kidney impairment by reducing inflammation, oxidative stress, and apoptosis. Kato et al. [25] studied the aldose reductase inhibitory (ARI) activity of several phytoconstituents in *Zingiber officinalis Roscoe* rhizome and discovered that they had good inhibitory action. The plant’s protective impact on diabetic kidneys, on the other hand, was characterized by the prevention of structural abnormalities caused by elevated free radicals after a brief period of hyperglycemia. Moreover, it helped to protect the renal system from membranous glycation-induced endothelial dysfunction.

### 3.2. Garlic

Garlic, also known as *Allium sativum*, belongs to the family of Amaryllidaceae. Due to its vast range of uses as a traditional spice and a key element in folk medicine prescriptions, garlic is considered an essential herb. A large amount has been learned regarding the various photochemical characteristics of garlic [26,27]. Many recent studieshave established garlic’s functional activity in cardiovascular disease [28,29] and cancer. It possesses anti-inflammatory, cardio-protective, anti-diabetic, antioxidant, reno-protective, anti-hypertensive, anti-microbial, immunomodulation, and antiviral effects [28,29,30].

According to a recent study by Arellano et al. [31], allicin’s anti-inflammatory mechanism is thought to be based on the reduction of NF-κB activation, which is involved in the synthesis of pro-inflammatory cytokines. Furthermore, allicin inhibits the activity of the NF-κB regulatory unit, which is essential for pro-inflammatory cytokine transcription activation and activation of TGF-*β*1 receptors. These inflammatory markers were found to be elevated in the diabetic group but controlled by treatment with allicin.

Allicin plays a role by inhibiting the signaling pathway of TGF-*β*1 and p-ERK1/2 production in HK-2 cells, which are dose-dependently stimulated by high glucose levels and are connected to epithelial myo-fibroblasts’ trans-differentiation. When compared with the allicin treated group and untreated diabetic control group, the latter showed elevated blood glucose levels, elevated levels of creatinine clearance, diuresis, elevated levels of glucose in urine, urinary excretion of N-acetyl-D-glucosaminidase (NAG), proteinuria, and also the initiation of free radicals and the expression of interleukin 1 (IL-1), nuclear factor kappa beta (NF-κB), interleukin (IL-6) and transforming growth factor beta-1 (TGF-*β*1). Treatment with allicin has been shown to reduce hyperglycemia, polyuria, and NAG excretion. This is mainly attributed to the antioxidant properties of allicin [32].

### 3.3. Cinnamon

*Cinnamon Cassia* has been a popular spice in several cultures around the world for many years. Other than its use for culinary purposes, it is recommended as a therapy for treatment of digestive, respiratory, and gynecological disorders. Various parts of the cinnamon tree, such as leaves, flowers, fruits, bark, and roots, have culinary and medicinal properties. The volatile oils extracted from the bark, leaves, and roots have a wide range of chemical compositions, implying that their pharmacological effects may vary as well. Active constituents of this plant include cinnamaldehyde, eugenol, and camphor. Cinnamon is mainly used in the treatment of cancer, inflammation, for its cardio-protective effects, as an antioxidant, to prevent migraines, as a treatment for Alzheimer’s disease, and for its anti-microbial activities [33,34].

Cinnamon and its procyanidin-B2 (PCB2) enriched fraction have good inhibitory effects in vitro, inhibiting the generation of AGE in diabetic nephropathic rats. Streptozotocin induced diabetic rats were given 3 percent cinnamon or 0.002 percent PCB-fraction for 12 weeks. At the end of the experiment, biochemical analysis of urine and blood had been performed. The renal biomarkers were evaluated using immunohistochemistry, immunoblotting, and reverse transcription polymerase chain reaction (RT-PCR) as measures of renal function. Suppression of glycation-mediated red blood cells-immunoglobulin G (RBC-IgG) cross-links and HbA1c augmentation in cinnamon and PCB2 treated diabetic mice was observed. It also reduced the significantly advanced glycated end product, N-carboxylmethyl lysine (CML), from accumulating in diabetic kidneys. Treatment with cinnamon inhibited the advanced glycation end product mediated decrease of the expression of glomerular podocyte proteins, including nephrin and podocin. Treatment with cinnamon and the PCB2 fraction reduced urinary albumin and creatinine, thus ameliorating renal malfunction. In conclusion, cinnamon reduced AGE formation in diabetic rat kidneys and improved DN pathogenesis. However, the protective effect of the plant on the diabetic kidney was characterized by the prevention of deprivation of nephrin expression. Nephrin is regarded as a tool for the function of glomeruli. Cinnamon was found to inhibit the kidney receptor for advanced glycation end products (AGE-RAGE), activated monocyte chemoattractant protein-1, and the expression of protein kinase C- *α* (PKC-*α)*, resulting in modulating the slit diaphragm proteins, that is expression of podocin and nephrin [35].

Because nephrin expression is thought to be a key measure of glomerular function, efforts to conserve it in clinical circumstances like DN show a lot of promise for treating a variety of kidney illnesses in which nephrin loss is a risk factor. According to the research conducted by Muthenna P et al. [10] RAGE may play a role in the regulation of nephrin expression. The results of this study showed that there was a decrease in proteinuria and podocyte injury in cinnamon treated rats. This impact could be attributable to the suppression of renal AGE-RAGE driven monocyte chemoattractant protein-1 (MCP-1) and PKC-α production, which modulates the expression of the slit diaphragm proteins nephrin and podocin. Cinnamon volatile oil, which contains 98%cinnamaldehyde, provided dose-dependent protection against alloxan-induced kidney damage.

### 3.4. Clove

Commonly known as (*Syzygiumaromaticum*) clove, this precious spice belongs to the family of *Mirtaceae* which has been used for centuries as medicine and as a food preservative because of its antimicrobial and antioxidant properties. It contains a variety of volatile oil in it. Caffeic, ferulic, salicylic, and ellagic acids are some of the phenolic acids present in clove. It also contains some flavonoids, including quercetin and kaempferol. The antioxidant activities of ethanol and water extracts of a different spices, including pepper, onion, cinnamon, garlic, mint, ginger, and clove, were investigated, and it was discovered that clove has the most antioxidant properties of all the spices tested [36,37,38].

Joshuva et al. [11] investigated a study on the evaluation of renal function of streptozotocin induced diabetic albino rats. The antioxidant property of clove and the active constituent present in clove, primarily eugenol, significantly reduces DN. This study shows that the clove extract reduced the glucose level and its complications, such as kidney disease (nephropathy), significantly when taken alone or in combination with metformin. This study also showed a significant difference in urea and creatinine. The kidney parameters in the treatment administered group, as compared to the positive diabetic control group significantly reduced, hence yielding a desired effect. (*p* < 0.0001). The findings from this same study also revealed that diabetic rats treated with clove had a 21% reduction in necrotic cells compared to diabetic rats. This study correlates with prior research in that it showed a regenerative effect when compared to the level of repair on Kupffer cells in the liver and kidney as compared to the diabetic group, which showed numerous cell differentiations and changes as revealed by histology. Eugenol has the potential to restore renal and liver function to closer levels by inhibiting lipid peroxidation and cytokine release.

### 3.5. Turmeric

Turmeric is a spice that has grabbed scientific and culinary attention, and it is a ginger related rhizomatous herbaceous perennial plant [39]. Most research concludes that the medicinal property of turmeric is mainly due to the active constituent named curcumin. It is basically grown in tropical and subtropical areas of the world and is mainly cultivated in countries such as India and China. Turmeric, also called curcumin, is the main natural polyphenol which is found in the rhizome of *Curcuma longa* (turmeric) and is called diferuloyl methane. It has a variety of medicinal properties, including treatment for diabetic wounds, rheumatism, anti-cancer, anti-hyperlipidemia, inflammation treatment, antimicrobial, anti-fertility, anti-venom, liver toxicity, renal injury, skin disease, and anti-platelets, among others. Due to the antioxidant, anti-mutagenic, anti-inflammatory, antibacterial, and anticancer characteristics, *Curcuma longa* has been used as a medicinal herb in Asian countries for centuries [40,41,42,43].

Lu M., et al. [12] investigated a study on curcumin, and it shows good DN inhibitory action. Curcumin Ameliorates DN by Suppressing NLR family pyrin domain containing threeinflammasome signaling (NLRP3). Curcumin’s reno-protective activity in streptozotocin induced diabetes nephropathy in rats has been established via lowering NLRP3 inflammasome signaling in lipopolysaccharide induced septic shock. Curcumin has been shown to reduce NLRP3 inflammasome activation and IL-1*β* (Interleukins-1*β*) production, and both IL-1*β* (a pro-inflammatory cytokine) and NLRP3 inflammasomes have been shown to be involved in the development of DN. Furthermore, recent reports have suggested that during acute and chronic kidney disorders, the NLRP3 inflammasome is the main influencer of inflammation and tissue damage [44].

Another study conducted by Sun LN et al. [41] on curcumin suggested that curcumin treatment inhibits DN through inhibiting the amountof gene expression. This action is by reversing caveolin-1 Tyr 14 phosphorylation that influenced Toll-like receptor-4 activation [45]. The research was mainly focused on the effect of treatment of curcumin on DN in db/db (diabetic) mice and its mechanisms on HK-2 cells. Findings from the study suggested that treatment with curcumin leads to a decrease in renal hypertrophy, improvement in renal function, and ameliorated renal histological alterations in db/db mice. The RT-PCR result of a study on curcumin reported that it may reduce DN progression by reducing the stimulation of the NLRP3 inflammasome [46].

According to a review of 14 randomized controlled trials by Wu W., et al. [47], there was a reduction in proteinuria, blood urea nitrogen, serum creatinine, and the mesangial area in rats treated with curcumin. Also, it protected kidney impairment in rats with diabetes. In another study, it was shown that curcumin considerably boosted AGEs, decreased superoxide dismutase activity in cell culture supernatant while greatly reducing AGEs raised malondialdehyde concentration. This result implies that curcumin has significant reno-protective activity [48].

### 3.6. Green Tea

Commonly known as *Camellia sinensis* and belonging to the Theaceae family, green tea is an ancient beverage with many therapeutic properties. It has anti-cancer, anti-inflammatory, anti-arthritic, anti-bacterial, anti-viral, anti-angiogenic, anti-oxidative, neuroprotective and anti-hyperlipidemic properties [49,50,51,52,53,54,55].

Green tea flavonoids have anti-inflammatory and anti-oxidative properties. This is due to the presence of polyphenols and flavonoids which protect the kidney from diabetes and hypertension-related renal oxidative stress.

In diabetic patients who had been receiving the highest recommended dose of renin-angiotensin system inhibitor, there had been a reduction in albuminuria after treatment with green tea polyphenol. This activity may be due to the inhibition of podocyte apoptosis via activation of the wingless-related integration site (WNT) pathway. The result of the study confirms in a clinical context that activating the WNT pathway and lowering podocyte apoptosis with green tea could reduce albuminuria in DN [56].

In a randomized clinical trial on 100 mildly hypertensive individuals, Mozaffari K., et al. [57] investigated the ameliorative effect of sour tea and green tea on blood pressure in patients with type II diabetes. They found that there was a drastic decrease in systolic and diastolic blood pressure in patients who were less hypertensive and consumed three glasses of green tea and sour tea daily for four weeks. Therefore, this finding suggests that the administration of green tea for hypertensive patients who are diabetic might protect the kidneys.

While glomeruli are the primary source of damage in DN, tubule interstitial alterations are extensively documented in people with type 2 diabetes [58,59]. On the other hand, proteinuria and DN development are closely linked to tubular damage in interstitial fibrosis [60]. As a result, it is acceptable to conclude that green tea has two distinct properties: an improved effect on metabolic and anthropometric indices and on hypertension, which indirectly saves the kidney from diabetic nephrotoxicity and protects the tubule through its antioxidant properties.

### 3.7. Guava

The guava tree (*Psidium guajava* Linn.), of the Myrtaceae family, primarily grows in tropical and subtropical regions. Its fruits and leaves have medicinal purposes, the fruit being used as a food source and processed into juice as well as jam [61]. Aside from these applications, Gutiérrez et al. [62] described the medicinal properties of guava, such as its use in liver disorders, allergy, its free radical scavenging property, its toxicity to normal cells, and its use for diabetes, cough, inflammation, cardiac disorders, pain, etc.

Lin C.Y., et al. [14] assessed the renal protective effects of guava aqueous extract (GAE) and ethanol extract (GEE) in diabetic mice by analyzing the concentration of phenolic acid and flavonoids in extracts of guava fruits. Myricetin, caffeic acid, and quercetin were present in higher quantities in GAE, and the results indicated that GAE reduced interleukin (IL)-6, reactive oxygen species, tumor necrosis factor, and IL-1 levels in the kidney. Fructose, N-(carboxymethyl) lysine, and pentosidine levels in the kidneys were reduced by 2% GAE and GEE treatments. These findings demonstrate that it has renal protective action through its anti-oxidative properties.

Many studies suggest that ferulic acid, ellagic acid, rosmarinic acid, and naringenin have been shown to have anti-diabetic properties [63,64,65]. Because these chemicals were also present in guava extracts, the existence of these extracts may have contributed to the observed protection. Overproduction of IL1, IL-6, and TNF has been shown to exacerbate diabetes worsening [66,67]. In diabetic mice, ellagic acid and naringenin have been shown to suppress the release of inflammatory cytokines from the kidneys [68]. As a result, the anti-inflammatory properties of guava extracts may be related in part to the action of these components. The findings of the above study suggested that there was suppression of DN by guava fruit extracts due to their anti-inflammatory and antioxidant properties.

### 3.8. Fenugreek

It is commonly known as *Trigonella foenumgraecum* L., belonging to the family Fabaceae, and is one of the oldest plants found in India and Northern Africa. Powders and extracts are prepared from its leaves and seeds, which have medicinal value. It has been used for bread production, as a supplement to wheat and maize flour, and as part of the general population’s daily diet [69,70]. Many pre-clinical and clinical studies have reported that extracts of fenugreek seed have been used to remedy anti-diabetic, hypo-cholesterolemic, and antioxidant properties. The ability of fenugreek seed powder (FSP) to reverse oxidative damage induced by oxygen-free radicals has been demonstrated as an indication of antioxidant enzymes, implying that it may have antioxidant properties. Because of diminished ROS generation and antioxidant enzyme stimulation, AGE production has been reduced and NF-kB has been activated. There is a significant reduction in IL-6 and inflammation in FSP treated diabetic rats, which could be associated with an elevation in antioxidant enzymes [71].

In FSP treated diabetic rats, there was a significant elevation in glutathione (GSH) concentration and a reduction in the malondialdehyde (MDA) level in the renal tissue. Furthermore, treatment with FSP leads to an elevation in the level of catalase and SOD. IL-6 and inflammation levels are remarkably reduced after treatment with FSP in diabetic rats, suggesting that it might be associated with antioxidant or anti-inflammatory potential. When NF-kB is inhibited, the expression of its regulated genes, such as IL-6, is suppressed. Blood glucose levels, ROS, interleukin-6, and DN were all elevated in the diabetic untreated group. The above results were confirmed with histopathology findings. The obtained data suggested that FSP may protect the kidneys from oxidative and inflammatory damage [72].

### 3.9. Gooseberry

Indian gooseberry or Amla is familiarly known as *Phyllanthus emblica* andbelongs to the family of Grossulariaceae. It is mainly grown in tropical parts of Southeast Asia. The fruit and its extract have been used for the treatment of diabetes, pain, cancer, obesity, constipation, and tumor treatment [73]. It is also commonly used as a gentle laxative.

Amla powder has a lot of phenolic antioxidants in it. According to the high performance liquid chromatography (HPLC) study, the ethanol extract of Amla contains a large amount of gallic acid, ellagic acid, and catechin hydrate. These natural antioxidants have the potential to effectively scavenge free radicals and reactive oxygen species (ROS). In sodium arsenate induced rats with renal disease, the 2K1C rats had considerably higher uric acid and creatinine levels than the control group in this investigation. After the treatment with amla powder, creatinine and uric acid in the plasma of 2K1C rats were normalized [74].

### 3.10. Oats

A high intake of whole-grain foods, such as oats, is mainly associated with a lower risk of cardiovascular complications and type-2 diabetes. Al Maliki et al. conducted an anti-diabetic study on oats. In this study, streptozotocin (STZ) induced diabetic rats developed DN as determined by elevated serum blood urea nitrogen (BUN), creatinine, creatinine clearance, and 24-h urine albumin. Oats supplementation for 21 weeks considerably improved renal function and had a hypoglycemic impact, which contributed to the reversal of DN [75].

### 3.11. Pepper

*Piper nigrum* belongs to the family Piperaceae and is the most prominent species of this genus. Piperine is also called the "father of spices.” It has beneficial health and disease-inhibiting properties and possesses antiviral, immunomodulatory, anti-inflammatory, anti-pyretic, and bioavailability enhancement [76,77,78].

The main alkaloidal phenolic component of black pepper is piperine, which possesses different medicinal uses, including antioxidant properties, and it activates digestive enzymes in pancreas and reduces lipid peroxidation.

Samra Y.A., et al. [17] described the study on the combination of cepharanthine and piperine on the progression of diabetes and concluded that animal groups after treatment showed a significant decrease in kidney weight. Serum creatinine was increased in the animal group treated with streptozotocin, which was significantly reduced after treating with piperine and cepharanthin and their combination. Piperine is an antioxidant that prevents lipid peroxidation and quenches free radicals and ROS, thus protecting against oxidative damage.

Cepharanthine, piperine, or their combination dramatically decrease lipid peroxidation and recover the drop in superoxide dismutase (SOD) levels. They also ameliorated inflammatory responses by suppressing NF-kB activation, resulting in lower levels of the inflammatory cytokines TNF-*α* and IL-1.

There was an increase in the level of thioredoxin interacting protein (TXNIP) and renal impairment in the rats that were induced with streptozotocin. Treatment with cepharanthine and piperine attenuated the renal damage induced hyperglycemia in diabetic rats.

### 3.12. Coriander

Commonly known as *coriandrum sativum* L., coriander belongs to the family of Apiaceae. The main phytoconstituents of coriander are alkaloids, flavones, tannins, resins, sugars, alkaloids, anthraquinones, and fixed oil sterols, [79,80]. The chief components of coriander fruit are essential oil and fatty oil. Fatty acids present in coriander include petroselinic acid (cis-6-octadecenoic acid, 18:1), linoleic acid (18:2), oleic acid (18:1), and palmitic acid (16:0). Like other green leafy vegetables, coriander is also a good source of vitamins, minerals, and iron, with the least saturated fat and cholesterol, in addition to zinc, thiamine, and dietary fiber [81,82].

Kajal A., et al. [18] conducted a study on *Coriandrum sativum* by administering the doses of 100, 200, 300, and 400/kg of petroleum ether extract of *C. sativum* (CPE) for 45 days. Biochemical parameters such as serum glucose, lipid and creatinine levels were reduced. Other parameters like advanced glycation end products formation, lipid peroxidation and thiobarbituric acid reactive species were significantly attenuated in the kidneys. A molecular docking system was used to understand the mechanism. Therefore, these results indicated that active constituents present in coriander might attenuate the further progression of diabetes nephropathy.

### 3.13. Silymarin

Silymarin increases protein synthesis and cellular regeneration in the kidney epithelium via stimulating ribonucleic acid (RNA) polymerase I. These effects are thought to be mostly caused by silybin, Lysiuk, and silichristin, while silidianin has little effect. According to the study, silymarin may be especially beneficial in cases when the renal epithelium is necrotic [83].

Silymarin bioflavonoid has antioxidant and anti-inflammatory properties, and it induces protein synthesis, suppresses lipid peroxidation, leukotriene and prostaglandin formation, and neutrophil migration [84,85,86]. Silymarin may have beneficial effects in the treatment of patients with renal insufficiency. Recent studies show that treating hemodialysis patients with silymarin alone or in combination with vitamin-E lowers plasma MDA levels while increasing blood glutathione peroxidase and haemoglobin levels [87]. In alloxan-induced diabetes rats, treatment with silymarin lowered the damage to the kidney and restored superoxide dismutase, catalase enzyme activity and glutathione peroxidase. Extract of milk thistle inhibits diabetic renal damage in streptozotocin induced diabetes rats, most possibly via elevating glutathione peroxidase and catalase activity and lowering lipid peroxidation in renal tissue [88].

A few studies have demonstrated silymarin’s potential efficacy in the treatment of DN. Fallahzadeh et al. [89] recently investigated the impact of adding silymarin to renin-angiotensin system inhibitors could reduce proteinuria in patients with type 2 diabetes with macro-albuminuria. The free radical scavenging and anti-inflammatory characteristics of silymarin have been thought to be correlated with a decrease in proteinuria. In diabetic kidney disease patients, silymarin was also found to lower albumin, TNF-*α*, and MDA excretion [89,90].

### 3.14. Tulasi

The scientific name of tulasi is *Ocimum sanctum* (family of Labiatae). This plant is also known for its medicinal properties. The medicinal herb is employed in indigenous medicine. Eugenol, carvacrol, urosolic acid, rosmarinic acid, linalool, *β*-caryophyllene, eugenic acid, geraneol, and ocimene are among the principal chemical elements in the aqueous extract of the leaves [19].

This plant possesses anti-stress, anti-asthmatic, anti-fungal, anti-bacterial, anti-oxidant, anti-viral, anti-tumor, gastric anti-ulcer activity, immunostimulant and anti-mutagenic activities. Additionally, these plants have been used to treat diabetes, cataracts, hypertension, diarrhea, cardiac toxicity, allergic hypercholesterolemia, depression, thyroid, neurotoxicity, and rheumatoid arthritis. Other medicinal properties include the chemo-preventive, anti-microbial, anti-inflammatory, radio protective, anti-carcinogenic, analgesic, anti-pyretic, memory enhancement, anti-tussive, anti-fertility, anti-emetic, anti-spasmodic, anti-stress, and the anti-coagulant [91].

The study proposes that *Ocimum sanctum* protects the kidneys through antioxidant and anti-inflammatory mechanisms like those found in angiotensin receptor blockers (ARBs) and statins. *Ocimum sanctum*’s antioxidant impact may help to minimize the formation of AGEs caused by diabetes. DN is associated with oxidative stress, AGE formation, extended hypoxia, iron accumulation, and inflammatory cell infiltration. Chronic hypoxia transforms tubular cells into myo-fibroblasts, hastening tissue fibrosis, which is exacerbated by breakdown of oxidative matrix proteins, the infiltration of inflammatory cells, and AGE modifications [92].

### 3.15. Red Sandal Wood

Belonging to the family Fabaceae, treatment with *Pterocarpus santalinus* resulted in a considerable reduction in blood sugar levels as well as an improvement in glucose tolerance tests. The extract of red sandalwood showed an antioxidant effect, as it reduced MDA levels. The extract also increased antioxidants, catalase superoxide dismutase and lowered lipid peroxidase synthesis, as determined by a thiobarbituric acid reactive substance. Serum creatinine and urine albumin were reduced after the treatment. The result was supported by histological report of the kidney for DN, and it indicated that combination therapy for 16 weeks indicated a reduction in levels of lipid profiles and an increase in the high density lipoprotein cholesterol of diabetes treated rats [20,93].

## 4. Clinical Trial Studies on Diabetic Nephropathy

Taghizadeh M., et al. [94] conducted a clinical trial to study the effects of mulberry extract affecting insulin metabolism indicators, lipid concentrations, and biomarkers of inflammation and oxidative stress in DN patients. Mulberry extract supplementation improved serum lipids, glutathione, and malondialdehyde levels in DN patients, but it had no effect on insulin metabolism indicators or biomarkers of inflammation and oxidative stress.

Vanaie et al. [95] aimed to determine whether curcumin could lower albuminuria in diabetic nephropathic patients. They concluded that curcumin is a safe adjuvant medication for type 2 diabetic patients with macroscopic proteinuria. Its effect can be shown after a short period of treatment (two months) and even in patients with a mild reduction in GFR. Curcumin is safe in normal doses; nevertheless, only long-term users have suffered peptic ulcers, gastrointestinal discomfort (GI), and a tendency to cause bleeding when taken along with anticoagulants.

Herbal therapies might create considerable health concerns when they postpone or replace a more effective form of standard therapies, or when they impair the potency of standard medicines. As a result, scientists feel that adding herbs to traditional medication therapy should be done with caution, considering the interaction or counteraction of each component [96,97]. The majority of herbal medicinal treatments have insufficient pharmacokinetic, pharmacological, and clinical data, which contributes to the lack of certainty of safety and efficacy. Future research aimed at identifying active components is the only realistic approach for substantiating efficacy claims for all herbs.

## 5. Summary

Nephropathy and cardiomyopathy are the major risk factors for diabetic patients worldwide, and their occurrence is increasing in the elderly population. Despite tremendous therapeutic inventions for diabetic complications, there is a lack of medical solutions with enhanced safety and efficacy, since modern medications being used to treat diabetes complications are correlated with several adverse effects. New treatment options are required to avoid oxidative stress problems and to enhance recovery. It is known that treating oxidative stress with antioxidants can effectively reduce diabetes complications. Hyperglycemia-induced oxidative stress is a critical factor in the development and progression of diabetic microvascular diseases like nephropathy [98].

Diabetes mellitus is an endocrine disorder that affects people of all ages. The main cause of alterations in kidney function in diabetes mellitus is persistent hyperglycemia. In hyperglycemia, inflammation and kidney injury cause an increase in advanced glycation end-products and activation of the polyol pathway, leading to oxidative stress. Endothelial cells, mesangial cells, and macrophages in the kidney produce more extracellular matrix proteins because of AGEs. Furthermore, it has been shown that the cross-linking of extracellular matrix proteins will reduce the matrix protein flexibility, which causes an imbalanced interaction with other matrix components [99].

Traditional medicine plays a very important role in the healthcare sector of many developing nations, such as India, and medicinal plants contribute significantly to this practice. According to the research, diabetes and its complications have become serious health issues in India. The current review explains that medicinal plants used for diabetes prevention and treatments have affected their therapeutic effects via a variety of pharmacological mechanisms. Ginger, for example, slowed the progression of diabetes nephropathy by lowering lipid peroxidation and increasing plasma’s antioxidant capacity. Most plants, including gooseberry, pepper, coriander, guava, and green tea, are used to treat DN due to their antioxidant properties.

More methodological reliability, clinical trials, validity, and suitable sample size are required to gather more definite data on the efficacy and safety of herbal extracts traditionally used in Indian medicine for treating diabetes and its complications.

## 6. Conclusions

According to the findings of this review, plants have a very varied lifestyle, and traditional remedies with antioxidant properties might be employed in the treatment of diabetes nephropathy, as the formation of stress and free radicals is a key cause of diabetes nephropathy. Since polyphenols, flavonoids, and essential oils have extensive antioxidant properties, we may infer in our article that the medicinal plants mentioned above can suppress diabetes and its complications with minimal side effects. Overall, phytochemical constituents from herbs may seem like a good and safe replacement, providing immense potential for research and discovery.

## Figures and Tables

**Table 1:** List of medicinal plants and their action on diabetic nephropathy.

Botanical Name	Family	Common Name	Parts Used	Mechanism of Action	References
*Zingiber officinale*	Zingiberacae	Ginger	Rhizomes	Lowers lipid peroxidation and enhances plasma antioxidant capacity	[8]
*Allium sativum*	Amaryllidaceae	Garlic	Bulb	The anti-glycation and hypolipidemic properties of aged garlic extract may be responsible for its protection against DN.	[9]
*Cinnamon cassia*	Lauraceae	Cinnamon	Bark	Prevents AGE accumulation in vivo and reduced alteration in the renal function of diabetic rats.	[10]
*Syzygiumaromaticum*	Myrtaceae	Clove	Roots	Decreases lipid peroxidation and cytokine release.	[11]
*Curcuma longa*	Zingiberaceae	Turmeric	Seeds	Curcumin’s protective impact in DN may be due to the suppression of NLRP3 inflammasome activation.	[12]
*Camellia sinensis*	Theaceae,	Green tea	Leaves	Green tea protects the kidneys from DN by preventing glomerular hyperfiltration, hypertrophic alterations, and protein loss in the urine.	[13]
*Psidium guajava*	Myrtaceae	Guava	Fruits	Anti-inflammatory, antioxidant and anti-gylcative properties.	[14]
*Trigonella foenumgraecum*	Myrtaceae	Fenugreek	Seeds	By reducing renal oxidative stress and inhibiting the TGF-β1/CTGF signaling pathway.	[15]
*Phyllanthus emblica*	Grossulariaceae	Gooseberry	Fruits	Gallotanin, an important ingredient of gooseberry, was found to be efficient in lowering plasma creatinine levels and reducing apoptosis by blocking poly ADP-ribose polymerase cleavage.	[16]
*Piper nigrum*	Piperaceae	Pepper	Fruit	Inhibited NF-κB and NLRP3 activation.	[17]
*Coriandrum sativum*	Apiaceae	Coriander	Seed	Delayed progression of DN, by inhibiting AGE.	[18]
*Ocimum sanctum*	Labiatae	Tulasi	Leaves	Antioxidant and anti-inflammatory activity.	[19]
*Pterocarpus santalinus*	Fabaceae	Red sandalwood	Bark	Antioxidant activity.	[20]

## Abbreviations

AGE—advanced glycosylated end products; ARB—angiotensin receptor blocker; ARI—aldose reductase inhibitors; BUN—blood urea nitrogen; CML—N-carboxylmethyl lysine; CPE—petroleum ether extract of *C. sativum*; DN—diabetic nephropathy; FSP—fenugreek seed powder; GAE—guava aqueous extract; GBM—glomerular basement membrane; GEE—guava ethanolic extract; GSH—glutathione; HPLC—high performance liquid chromatography; IL-6—Interleukins 6; IgG—immunoglobulin G; LDL—low density lipoprotein; MCP-1—monocyte chemoattractant protein-1; MDA—malondialdehyde; NF-κB—nuclear factor kappa B; NLRP3—NLR family pyrin domain containing 3; PCB2-procyanidin-B2; PKC—protein kinase C; RAGE—receptor for advanced glycation end products; RBC—red blood corpuscles; RNA—ribonucleic acid; ROS—reactive oxygen species; RT-PCR- reverse transcription–polymerase chain reaction; SOD—superoxide dismutase; STZ—streptozotocin; TC-total cholesterol; TG—triglyceride; TGF--*β1*-transforming growth factor beta one; TNF-*α*—tumour necrosis factor alpha; TXNIP—thioredoxin interacting protein; WNT—wingless-related integration site.
